# Management of Pediatric Mandibular Condyle Fractures: A Literature Review

**DOI:** 10.3390/jcm13226921

**Published:** 2024-11-17

**Authors:** Gian Battista Bottini, Fabio Roccia, Federica Sobrero

**Affiliations:** 1 Department of Oral and Maxillofacial Surgery and Center for Reconstructive Surgery, University Hospital of the Private Medical University Paracelsus, 5020 Salzburg, Austria; 2 Division of Maxillofacial Surgery, Città Della Salute e Della Scienza Hospital, University of Turin, 10126 Turin, Italy; fabio.roccia@libero.it (F.R.); federica.sobrero@unito.it (F.S.)

**Keywords:** mandibular condyle fractures, pediatric mandibular condyle fractures, pediatric maxillofacial trauma, expectant management, maxillomandibular fixation, intermaxillary fixation, functional orthodontic therapy, open reduction and internal fixation

## Abstract

This narrative review evaluates the literature on the management of mandibular condyle fractures in growing patients. It aims to illustrate some fundamental biological principles and to offer a series of considerations applicable to clinical practice. The discussion is based on 116 papers published in PubMed and two relevant textbooks. Condylar fractures may be overlooked, especially in pre-scholar children, where compliance is usually reduced. However, these injuries can have disabling sequelae such as ankyloses, facial deformities, malocclusion, and chronic pain in some patients if not diagnosed and managed correctly. Due to their significance, mandibular condyle fractures in children are a subject of considerable clinical interest. As of today, there is consensus about their treatment. Four management options are available: expectative (analgesia, soft food and follow-up), functional protocols (guiding elastics, orthodontic appliances and exercises), maxillomandibular fixation (MMF), and open reduction and internal fixation (ORIF). Nondisplaced and minimally displaced fractures should be treated expectantly; severely displaced non-comminuted fractures can be safely operated on if the expertise is available, even in patients with deciduous dentition. Moderately displaced fractures can be managed with functional protocols or operatively, depending on the background and know-how of the specialist. Functional protocols can achieve good outcomes, especially in patients with deciduous dentition. MMF should be foregone in children due to its many drawbacks.

## 1. Introduction

Pediatric maxillofacial fractures comprise up to 15% of all maxillofacial fractures in children and adults [1,2,3,4]. Fractures in children under five years old are uncommon and mainly caused by falls. Along with falls, other causes are road traffic accidents, sports, and assaults [3,4,5,6]. Mandibular fractures account for 20 to 50% of pediatric facial fractures, with the condylar process frequently affected [2,3,5]. Condylar fractures may be overlooked, especially in pre-scholar children, where compliance is usually reduced. However, these injuries can have disabling sequelae such as ankylosis, facial deformities, malocclusion, and chronic pain in some patients if not diagnosed and managed correctly [5,7,8,9]. Due to their significance, mandibular condyle fractures in children are a subject of considerable clinical interest. Four management options are available: expectative (analgesia, soft food and follow-up), functional protocols (guiding elastics, bite-rising appliances and exercises), maxillomandibular fixation (MMF), sometimes called “closed treatment” to contrast it with open surgery, and open reduction and internal fixation (ORIF).

There is no agreement on the best possible treatment for mandibular condyle fractures in children, which can create uncertainty in the care providers, given the importance of achieving the best possible outcome and avoiding sequelae in children [3,7,8]. A recent multicentric retrospective study on the management of pediatric condylar fractures highlighted the preference for expectative, closed and operative treatment across 14 maxillofacial departments worldwide, whereby functional protocols were not implemented, and the choice of management appeared to be based on the local teaching and training and the surgeon’s expertise [10].

Irrespective of the protocol, the goal is to regain pain-free temporomandibular joint function with a full range of motion, pre-injury occlusion, facial balance, and expected growth.

The present paper aims to illustrate some fundamental biological principles and offer a series of valuable considerations in clinical practice.

## 2. Materials and Methods

A narrative review of the literature published on PubMed and of relevant textbooks in the English and the German language was conducted. A systematic review is appropriate to answer a focused question. On the other hand, a narrative review is better suited for providing an overview and linking other topics to the main one with a greater degree of freedom. As the literature on pediatric mandibular condyle trauma consists mainly of retrospective series from single institutions, the literature search was extended to the adult population to include studies deemed necessary or proper for the discussion. The database, PubMed, was searched using the words “pediatric maxillofacial trauma”, “pediatric mandibular condyle fractures”, and “mandibular condyle fractures”. Case reports and animal studies were excluded. After reading the titles and the abstracts, full texts of relevant articles were analysed. The search was expanded manually to include the significant papers cited in the above articles. Pertinent textbooks in English and German were consulted.

## 3. Results

The literature search yielded 116 articles and two textbooks [11,12] dealing directly with or related to pediatric condylar fractures and their management.

## 4. Discussion

### 4.1. Fractures Classification and Differences Between the English and German Terminology

In the present review and their clinical practice, the authors refer to Loukota’s condylar fracture classification and subsequent modification by Neff et al., which divides these fractures according to their anatomical level (condylar head, neck, or base) [13,14].

These fractures can be further characterised based on their type: nondisplaced, displaced, comminuted or dislocated. Confusingly, the English and the German terminology use different words for the same fracture types, whereby a fracture with exarticulation from the joint is a dislocated fracture in the English literature and a “Luxationsfraktur” (lit. Luxation fracture) in the German literature [11,15]. A fracture with a separation of the fractured segments is called displaced in English literature and “disloziert”(dislocated) in German literature [11,15].

According to their dentition stage, pediatric patients are often divided into three groups: deciduous (0–5/6 years), mixed (6–11/12 years), and permanent dentition (12–16 years).

### 4.2. Management Options

Four management options are available for mandibular condyle fractures: 1. expectative (analgesia, soft food and follow-up), 2. functional protocols (guiding elastics, bite-rising appliances and exercises), 3. maxillomandibular fixation (MMF), often termed “closed treatment” in opposition to open surgery, and 4. open reduction and internal fixation (ORIF). Below, we describe the above four options in more detail, discussing their advantages and disadvantages and illustrating some fundamental biological principles that constitute the rationale for their implementation.

### 4.3. Expectative Management

Bones in children are more elastic than in adults, as they are not yet fully mineralised. Therefore, they have a greater capacity to deform and buckle without fracturing [16,17]. Because of its superior elastic characteristics, pediatric bone can tolerate tension better than compression. Fractures in children are often incomplete. Therefore, the periosteum maintains its gross integrity, and the bone is not separated, similar to a break in a slender willow branch (“greenstick fracture”) [18]. The periosteum, with its inner cambium layer which is rich in skeletal progenitor cells, osteoblasts, and blood vessels, is crucial in fracture healing and can regenerate cartilage and bone [19,20]. The above incomplete and favourable fracture morphology is coupled with a much higher osteogenic capacity of the pediatric periosteum [18]. The bone in children is also more richly vascularized as it is mainly made of spongiosa with a very thin cortical layer, ensuring a rich flow of cells for the repair process and increased metabolism [21].

Moreover, the bite force of children is much smaller than that of adults [18]. Crucially, the mandibular condyle process is a growth centre with an incredibly potent regeneration capability if its growth still needs to be completed. Therefore, it is possible to achieve fracture healing with restitutio ad integrum without immobilisation. All authors agree that nondisplaced or minimally displaced mandibular condyle fractures do not need any active treatment. Adequate analgesia (e.g., paracetamol or non-steroidal anti-inflammatory drugs) with a liquid diet followed by pureed and soft food will ensure patients’ comfort and adequate nutrition. The movement will expedite oedema resolution and healing.

At the first author’s institution, weekly follow-up clinical examinations are carried out in the first 4–6 weeks, and a steady improvement in range of movement until reaching full range of motion is to be expected. Clinical examination and imaging are performed at 6 and 18 months after the trauma, and thereafter, a yearly follow-up is advised until growth completion to exclude asymmetries and ankylosis.

### 4.4. Functional Protocols and Outline of Theories on Craniofacial Development

Even in the absence of immobilisation, the extraordinary healing capacity of the pediatric mandibular condyles defies one of the basic tenets of fracture treatment. Somewhat paradoxically, immobilisation of these fractures can worsen the outcome for these children.

Bone remodelling is the process by which living bone, subject to the functional demands of the muscles and soft tissues, changes its shape, density, and position. Moss famously postulated that muscular loads on bone determine craniofacial development (functional matrix hypothesis) [22]. “The developmental origin of all cranial skeletal elements (e.g., skeletal units) and all their subsequent changes in size and shape (e.g., form) and location, as well as their maintenance in being, are always, without exception, secondary, compensatory, and mechanically obligatory responses to the temporally and operationally prior demands of their related cephalic nonskeletal cells, tissues, organs, and operational volumes (e.g., the functional matrices)” [22]. “More precisely, the functional matrix hypothesis claims that extraskeletal factors and processes are the prior, proximate, extrinsic, and primary cause of all adaptive, secondary responses of skeletal tissues and organs” [22]. As discussed above, at the tissue level, the periosteum—soft tissue—regulates bone growth. At the cellular level, a load can generate intracellular electrical or chemical signals (mechanoelectrical and mechanochemical transduction), thereby influencing osteocytes and osteoblast activity, with consequent bone resorption or deposition. (Moss1) A detailed description of the molecular mechanisms involved in these processes exceeds the scope of this review. The interested reader is referred to Moss’s papers [22,23,24,25].

Following Moss, Enlow also explained craniomaxillofacial development as the result of the interaction of bone displacement due to the surrounding soft tissues (action) and consequent bone remodelling (reaction) [26]. Bone displacement was defined as the separation of bones at sutures, joints, and synchondroses, like in cranial vault expansion due to brain growth [26]. The areas of pressure with bone resorption at the inner table (endosteum driven) and the areas of tension with bone deposition at the outer table (periosteum driven) result in cranial vault expansion or circumferential growth in a long bone [26].

Scott, a proponent of the “cartilaginous theory”, distinguished between primary cartilage (cranial base and septum) and secondary cartilage (condyle) [27]. The mandibular condyle is not a “driver of growth” primarily genetically driven like the cranial base and the nasal septum. It is instead a secondary growth centre “adaptive in nature” in that the condylar head cartilage “does grow and ultimately ossify, but it does so in response to function and is under significant environmental influence” [27,28,29]. Contraction of the lateral pterygoid muscle “stimulates mitosis of the prechondroblasts and compensatory ossification of the growth plate” [30].

Therefore, a functional protocol in a displaced condylar fracture can enhance condylar remodelling in children.

The above is the rationale for early mobilisation and prolonged physiotherapy, practising passive and especially active mouth opening, protrusion, and lateral excursions. Thorén, in her group of patients with condyle dislocation from the glenoid fossa, concluded her thorough analysis suggesting the following: “Soft diet, early mobilization, and close observation of the occlusion is the treatment of choice. However, in cases of primary open bite, guiding elastics that allow mobilization should be used” [31].

The capacity to remodel the medially displaced condylar fragment back to a shape and position identical to the pre-injury (complete remodelling) is gradually lost towards the end of the growth. Therefore, a displaced fractured condyle in a skeletally mature patient will heal in malposition and remain deformed [32,33]. Chang et al. demonstrated complete condylar remodelling in children with a series of serial 3D reconstructions, whereby the reshaping and straightening of the condylar process is the result of concomitant bone reabsorption on one side and bone apposition on the opposite side of the proximal fragment [32]. Yamashita et al. showed similar 3D reconstructions in adult patients, whereby dislocated proximal fragments heal in malposition. They do not resume their previous shape or straighten themselves but remain bent and deformed [32,33].

The condylar remodelling capacity is at its maximum in the first years of life up to the age of approximately 5 years, but between 6 and 12 years, it decreases, and, after 12 years, it tapers down to the adult level at the end of skeletal growth [34].

Zhu et al. performed a three-dimensional evaluation of condylar morphology after closed treatment in intracapsular condylar fractures in growing patients, measuring 29 landmarks and eight parameters on 3D skull models, reporting complete remodelling with restitutio ad integrum in children under six years [34]. On the other hand, children who were six or older healed with shortening and thickening of the fractured condylar process. However, the shorter condyle was compensated for by thickening the glenoid fossa and increasing ramus height so that the pogonion did not stray from the midline and symmetry and function were preserved [34]. Choi reported the same finding of the “flattening of the mandibular fossa” as a compensatory bone growth after condylar fractures with shortening of the condyle in children [35].

Beyond enhancing condylar remodelling in children, guiding elastics, often prescribed also in adult patients, have the additional purpose of steadying mandibular motion and guiding it into occlusion. In this case, the rationale is to facilitate neuromuscular rehabilitation by creating a neurologic engram by repeating the same movement pattern. In this respect, guiding elastics are comparable to orthopaedic tutors that do not block movement but rather support it. Therefore, the term “IMF with light elastic”, often encountered in the literature, is a contradiction and should be replaced with functional treatment with guiding elastics, as already noted in previous papers [36].

Children have lower bite force and higher osteogenic capacity than adults. Therefore, they can heal much faster than adults but also have a higher risk of ankylosis with a risk of long-term functional restrictions and corresponding patient’s burden [18,30,31,37,38].

Therefore, guiding elastics should be kept only as long as necessary to fulfil the purpose of occlusal guidance and removed as early as possible.

Practice shows that light guiding elastics suspended to orthodontic brackets bonded to the canines for a few weeks do not cause significant dental extrusion.

Different rules apply to managing pediatric mandibular condyle fractures than those for adult patients [32].

However, guiding elastics are not the only form of functional therapy.

Another option is represented by “activators” or “bionators”, a group of orthodontic appliances with different designs originally devised to “activate” the masticatory muscles and, as a result, to influence and correct mandibular growth and bite anomalies in children [12].

Their therapeutic effect is attributed to the stretching of the masticatory muscles, which determines bone and cartilaginous apposition in the condylar process and the mandible [22,30].

Nevertheless, when an activator has a wedge-like shape, higher in the front and thinner at the back, it is counterproductive when treating condyle fractures. This type of design reinforces the natural tendency to develop an open-bite malocclusion in condylar fractures with a shortening of the ramus (compression) [39]. Activators designed for skeletal discrepancies are generally contraindicated for treating condylar fractures with dislocation [39].

On the other hand, bite plates in the molar area on the fractured side or bilaterally in bilateral fractures have the effect of depressing the occlusal mandibular plane, creating space for the condylar process to straighten itself up (distraction) [39]. Adjuvant straight elastic IMF in the molar area on the healthy contralateral side in monolateral fractures, or at the front in bilateral fractures are helpful to counter the open-bite malocclusion [39]. The same principle of “hypomochlion”, “molar fulcrum”, “spacer”, or “bite-block” has also been described by Ellis in adult patients and by Zhao et al. in children without elastic IMF [38,40]. Wu et al. used resin to raise the bite posteriorly and adjuvant elastic traction via IMF screws [41].

Chen reported on a group of 189 pediatric patients with 273 condylar fractures where only one patient underwent ORIF, and the remaining 188 were managed with a soft diet, exercise and a removable occlusal splint [6].

Based on the remodelling capacity of the condyle, Li et al. have treated most pediatric patients with a functional protocol. A full-arch occlusal splint raised with self-curing resin in the molar region of the fractured side and the guiding elastics anchored to four intermaxillary screws were implemented for all their patients with condylar head and condylar neck and base fractures with mild displacement [42]. ORIF was reserved only for condylar neck and base fractures with severe displacement, achieving good functional results in surgical and nonsurgical cases [42].

Of note, a full-arch occlusal splint prevents dental extrusion of the teeth without coverage, which may happen in the medium term in the case of isolated posterior bite plates [38,42].

Therefore, a full-arch bite raised posteriorly with resin is appropriate to avoid unwanted dental extrusion if worn beyond the short term. As discussed previously, it can be coupled with guiding elastics in children with mixed dentition. Mandibular movement should not be restrained but encouraged and practised systematically as physiotherapy to achieve quicker and complete rehabilitation.

Malinge described a functional treatment based on Delaire’s technique consisting of “immediate active mobilization of the mandible with projection and lateral excursion movements” in 108 children (<15 years of age) with dislocation of the condylar process out of the articular fossa, leading to “satisfactory long-term functional and architectural outcomes” in most of the 33 patients that could be followed up until growth completion [30].

Du et al. contributed to shedding light on the remodelling capacity of the condyle in pediatric patients reviewing 3D reconstructions based on computed tomograms of 294 children and adolescents who underwent nonoperative treatment in the forms of natural healing (expectant protocol) or a maxillary splint with 5 mm pads on the last molar of the affected side with two weeks of anterior elastic IMF—depending on the dislocation degree—followed by physiotherapy [43].

Landes et al. used guiding elastics suspended to intermaxillary screws for two weeks in children 12 years old or older and a removable orthodontic appliance for three months in children younger than 12 to avoid injury to the teeth buds in mixed dentition [44]. For both groups, physiotherapy with active jaw movement was started in the third week and continued for two weeks [44].

Lekven et al. treated their pediatric patients with either observation or intermaxillary fixation using wire or guiding elastics. The clinical result was classified as either favourable or not. Based on bidimensional imaging, condylar remodelling was categorised as complete, moderate, or poor. A total of 74% of the patients [32] had a satisfactory long-term clinical outcome, while 26% [13] did not [45].

Sabbagh et al. assessed the effect of functional orthodontic treatment of mandibular condyle fractures in eight children and adolescents with clinical, functional, and magnetic resonance imaging. Scans showed condylar remodelling and uprighting of the condyles in most cases, achieving complete disc reposition [46]. They used an original spring activator designed to treat patients with open bites. The loop springs placed dorsally in the molar area caused a controlled distraction of the condyle instead of compression during temporal and masseter muscle activation, allowing the simultaneous stabilisation of the occlusal plane and jaws relationship as well as the distraction and mobilisation of the condyle, thereby providing advantageous prerequisites for remodelling [46]. Given the brilliant outcomes, they advised considering functional therapy when choosing nonoperative treatment for condyle fractures in children and adolescents. On the other hand, they lamented that maxillofacial surgeons rarely refer patients to the orthodontist, even in conservative treatment, thereby depriving the patients of a noninvasive, well-established and effective treatment [46].

Most of the authors favour nonoperative treatment in case of malocclusion for children until six years old and reserve ORIF for exceptional circumstances such as dislocation of the condyle in the middle cranial fossa, presence of a foreign body, severe malocclusion, or if the dislocated proximal fragment impairs the mouth opening [2,5,9,18,40,47,48,49,50].

There is consensus that functional treatment, not only as a stand-alone treatment but also after ORIF, is crucial to quickly achieving a complete range of motion [51,52].

### 4.5. Maxillomandibular Fixation

Many agree that the term “closed reduction”, frequently encountered in the literature, is a misnomer [40,51,53]. One could consider it an example of wishful thinking: no displaced or dislocated condylar fracture can be reduced without direct manipulation of the proximal segment. In dislocation, the proximal segment is typically displaced in an anteromedial direction by the pull of the lateral pterygoid muscle out of the joint. Repositioning of the proximal segment is usually possible during open surgery with direct manipulation under general anaesthesia and with complete muscular paralysis. The reduction can be reliably stabilised only with stable osteosynthesis. The only example of an actual closed reduction of displaced or dislocated pediatric mandibular condyle fractures has been reported by Kim et al., who ingeniously used a Kirschner wire and external rubber traction under fluoroscopic control to accomplish this goal [54]. Therefore, the term “closed reduction” should be abandoned and replaced by the more general term “closed treatment” to highlight the contrast with open surgery. However, it should be noted that the term “closed treatment” has been used for different approaches, such as intermaxillary fixation (IMF) and light guiding elastics. IMF or maxillomandibular fixation (MMF) with wires or heavy elastics is intended to immobilise the mandible—and the fracture—like a cast. Light guiding elastics instead are a form of functional treatment. Therefore, to eliminate confusion in communication, one should describe what means is being used to achieve IMF and, in the case of elastics, the type of force they exert, whether light or heavy.

An actual IMF has several drawbacks for children and adults: cumbersome in its application, risk of tooth injury, periodontal injury, risk to the airways, compromise of oral hygiene, malnourishment, limited range of motion after its removal, risk of ankylosis, discomfort, pain, anxiety, and poor compliance [29,38,55,56]. “Active movement of the jaw is significant in combating ankylosis in this highly vascularized and osteogenic environment” [37].

Thorén conducted a rigorous follow-up study of children with total dislocation of the condyle from the glenoid fossa and concluded that “no benefits were gained in our patients by using MMF” [31].

Ellis observed that active and passive movement of an injured joint promotes healing and rehabilitation, whereas “immobilization of a damaged joint leads to degeneration of the articular surfaces and development of fibrous adhesions, limiting mobility” [40]. He added that “studies in the animals support the finding that condylar process fractures heal irrespective of whether they are immobilized.” [40]. To the following question: “Is MMF necessary/desirable?” he concluded, “there is no compelling reason to use MMF when treating fractures of the condylar process by closed techniques” [40].

IMF in children with condylar fractures is not advised for its many drawbacks and the risk of ankylosis [38,55,56]. Zhao reports pain relief in their 40 pediatric patients when wearing a removable full-arch maxillary splint raised in the molar region due to its distraction and decompression of the TMJ on the side of the condylar fracture.

Some authors used MMF with wiring techniques [57,58]. However, this approach is unnecessary and obsolete. Dimitroulis claimed that IMF has an antalgic effect due to immobilisation. However, the present study’s authors believe that IMF determines compression at the fracture site and worsens the pain in the temporomandibular joint (TMJ).

Aksoyler and colleagues managed medially displaced sub-condylar mandibular fractures in children with a hybrid protocol [59]. Via a transoral incision under general anaesthesia, they reduced the proximal fragment, obtaining confirmation via three-dimensional intraoperative imaging, without plating the reduced fracture but stabilising it through intermaxillary fixation for two weeks, followed by physiotherapy for up to twelve weeks [59]. Their method has the advantage of being less traumatic compared to osteosynthesis and does not require a second intervention for hardware removal. However, the stability of the fixation is not guaranteed, and the patients must endure two weeks of IMF.

Because of its drawbacks, true jaw (and joint) immobilisation should be foregone in pediatric patients. On the contrary, light guiding elastics and active mandibular movement are crucial in promoting functional rehabilitation and condylar remodelling in children, enhancing their healing in cases with less severe dislocation [30,34].

Rozeboom carried out a systematic review on the closed treatment of unilateral mandibular condyle fractures in adults and reached the same conclusions, recommending expectant treatment in case of acceptable occlusion or guiding elastics in case of malocclusion, and never immobilisation of the jaw, even in adult patients [60]. Neff et al. observed that even in adults, in the case of nonsurgical treatment of condylar fractures, MMF is forgone by experts in the field and replaced by immediate functional training [47]. In conclusion, if the occlusion is acceptable after a condylar fracture in children, an expectant protocol has proven successful, and MMF is neither necessary nor indicated.

On the other hand, in case of malocclusion after a condylar fracture in children, functional treatment with a full-arch splint raised in the molar region and possibly additional guiding elastics is a minimally invasive option that is viable and effective.

### 4.6. Open Reduction and Internal Fixation

The approach to moderately and severely displaced or dislocated pediatric condylar fractures is controversial. It is striking to observe a diametrically opposite attitude: on the one hand, some authors treat every case with a conservative protocol; for others, ORIF is the default option for displaced and dislocated fractures, even in young children, to repair the bone and the soft tissues [61].

Despite acknowledging ORIF’s superiority for moderately and severely displaced and dislocated fractures in adults, English-speaking authors are against ORIF and prefer nonoperative treatment for treating the same fractures in children.

The justifications in favour of nonoperative treatment are the following: technical difficulties, reduced bone stock for plating, the risk of facial nerve injury, concerns of avascular necrosis and growth restriction due to the scarring that follows surgery (similarly to reduced maxillary projection after lip surgery in cleft patients) and the possibility that plates and screws may damage growth centres or hamper growth across sutures (similar to children with craniosynostoses), hardware translocation, disruption of the periosteal vessel and bone vascularity following periosteal elevation, the need for hardware removal [18,26,62,63], and several publications concluding that satisfactory functional results are obtained by nonoperative treatment thanks to the efficacy of condylar remodelling in children [15,18,47].

Regarding whether ORIF for condylar process fractures is biologically sound in adult patients, Ellis concluded that after repositioning with open treatment, less adaptation within the masticatory system will be required compared to nonoperative treatment in case of displacement [40]. However, in the same article, Ellis agreed that the nonsurgical approach is the default solution for children [40].

Naik found that adult patients treated with ORIF reported less pain and had better function compared to patients treated with intermaxillary fixation [64].

Madadian determined that ORIF is superior to nonoperative methods in adults with severe displacement or dislocation, offering superior functional outcomes in most cases [65].

A meta-analysis by Al-Moraissi and Ellis concluded that ORIF is superior to closed treatment in adults [66].

Bera and colleagues performed a meta-analysis of closed versus open versus endoscopic management for condylar fractures in adults, excluding the management of pediatric fractures as very controversial. They concluded that, in the adult, closed and open reduction “is equally effective in diacapitular fractures and minimally displaced neck and base fractures. Moderate to severe displacement with considerable height shortening warrants open reduction. Endoscopic approaches are associated with considerable technical challenges, and efficacy over open reduction is still not validated, dictating its use only in selected cases” [67].

McGoldrick et al. performed a retrospective analysis on pediatric condylar fractures, reporting stable outcomes with conservative management and suggesting that the treatment for these cases should be as conservative as possible [68].

Zachariades analysed 466 patients (adults and children) with mandibular condyle fractures [62]. They never treated surgically condylar intracapsular fractures, neither in adults nor in children [62]. Fractures in children until early teens were generally managed without surgery, regardless of the level and the type of condylar fracture [62]. They preferred closed reduction with or without one to three weeks of IMF, followed by guiding elastics and physiotherapy [62]. They claimed that, even in case of deviation, severe displacement or dislocation, the condylar process remodels recovering nearly normal anatomy and function and reserved surgery before puberty only for exceptional cases such as projectile injuries, major dislocations and polytrauma given the enhanced healing capacity and the technical difficulties of open surgery in small children [62]. Of note, their references appear to be outdated and all in favour of conservative treatments.

Notably, much of the literature on facial trauma from the USA is not written by maxillofacial surgeons but rather by plastic surgeons or ear, nose, and throat specialists who deal with facial trauma regularly.

Singh et al. indicated ORIF instead of conservative treatment only for adults with moderately or severely displaced condylar base fractures [69]. They claimed that condylar head and neck fractures should not be treated surgically because of the high risk of avascular necrosis [69].

Haug et al. agreed that even in adults, intracapsular fractures “should not be opened”, regardless of the type [50].

Jenkyn et al. conducted a systematic review on managing mandibular condyle fractures in pediatric patients to compare surgical and nonsurgical management. They could not discover “convincing evidence for the superiority or inferiority of surgical management of paediatric mandibular condyle fractures”, recommending “nonsurgical as first line with surgical being reserved for specific types of condylar fracture” as in severe displacement [70]. Of note is that their literature review was limited to the English language only.

Kommers et al. conducted a survey to assess the level of agreement on the classification and treatment decisions for three different types of condylar fractures in adults among 491 maxillofacial surgeons worldwide [71]. Surgeons from North America chose to perform ORIF significantly less often than surgeons from other parts of the world. European surgeons preferred expectative treatment significantly more often than IMF compared to their colleagues on other continents [71].

Deleyiannis and colleagues conducted a retrospective analysis of children treated with ORIF for dislocated condylar fractures. They removed the proximal dislocated fragment to plate it extra corporally and reinserted it as a graft in four out of six cases [72]. One of the patients required a secondary costochondral graft after near-complete resorption of the condyle [72].

The condyle receives blood from three sources: a branch of the inferior alveolar artery through the condylar neck, the temporomandibular joint capsule with its vascular plexus, and branches from the lateral pterygoid muscle [40]. Severing the last remaining vascular pedicle of the proximal fragment in case of severe displacement or dislocation will likely cause condylar bone reabsorption due to avascular necrosis (first iatrogenic injury) [40,51,72].

Importantly, in this situation, the osteosynthesis material, which is not fixated to the proximal fragment at its cranial extremity but still fixated to the distal segment, will probably traumatise the surrounding structures such as the capsule, the disc and the condylar fossa, with each mandibular movement potentially eroding the skull base (second iatrogenic injury).

Lopez and colleagues conducted a retrospective study of pediatric patients with mandibular condyle fractures [73]. They implemented a hybrid protocol in the form of open reduction without internal fixation, combined with intermaxillary fixation for three or even four weeks, and registered cases of ankylosis, malocclusion, malunion and mental nerve dysesthesia. Of note, no patient was treated with open reduction and internal fixation [73]. The authors of the present review disagree with the above protocol as no reliable fixation can be achieved without osteosynthesis, and intermaxillary fixation carries the risk of ankylosis and several other drawbacks, as discussed previously.

Yesantharao et al. focused on the treatment of combined symphyseal and condylar fractures in growing patients [74]. Condylar fractures were always treated conservatively; only symphyseal and parasymphysis fractures were treated surgically, and IMF was kept for three weeks [74]. The overall frequency of complications was high and was attributed mainly to deviation from their institutional protocol [74]. They recommended IMF with skeletal wiring for two weeks in children with deciduous dentition and displaced symphyseal fractures, followed by three to four weeks of elastics. In the mixed dentition, they preferred IMF for two weeks, followed by three to four weeks of elastics. Finally, in the permanent dentition, ORIF was used for the symphyseal fracture, followed by IMF for two weeks [74].

They explained malocclusion, mandibular growth disturbance and maleruption of teeth by the presence of fixation plates, claiming that “invasive manipulation of mandibular fractures may have adversely impacted these patients’ occlusal and dental development”. Furthermore, they suggest that “the functional matrix theory of pediatric craniofacial development states that children who undergo periosteal and soft tissue manipulation are more susceptible to growth deformity and complications such as malocclusion, as we saw in our data set” [74]. The authors of the present review disagree with their interpretation and attribute the above complications to the suboptimal ORIF technique, not to the ORIF per se, and to the fact that displaced condylar fractures were not repositioned. Yesantharao et al. explained their complications with the adverse effects of scars on growth or the lack of adherence to their institutional protocol. However, there is plenty of evidence that expert surgery does not hinder growth. Furthermore, they suggested using IMF with wires in children with deciduous dentition, which has several drawbacks and is considered by all experts obsolete and counterproductive. Finally, they did not operate on condyles, only on symphyseal fractures; therefore, the malocclusion cannot be improved if the condylar fracture is dislocated. Overall, their approach needs to be revised to a contemporary standard of treatment.

Yadav and colleagues assessed the condylar remodelling in their pediatric population, with mandibular condyle fractures treated by nonoperative management. Their premise was that the treatment for these cases should always be conservative and never open. They observed complete remodelling and satisfactory outcomes in their sample. However, they used exclusively panoramic X-rays to assess the condyle, and this is inadequate nowadays [35,43]. CTs and the 3D reconstructions represent contemporary imaging of condylar fractures. Furthermore, they stated that “there is no availability of high-level evidence for improved results with surgical management” and that “review of the literature supports nonoperative management for pediatric mandibular condylar fractures” [75]. The authors of this review disagree as there is enough evidence to say that ORIF of condylar pediatric mandibular fractures can achieve excellent function and remodelling. Yadav and coll. concurred with the great majority of the Anglo-American literature that condylar fractures in children should always be treated conservatively. Furthermore, their quality control was based on bidimensional imaging, which is inadequate nowadays to assess the healing of a condylar fracture. Finally, they did not refer to the German literature that reported successful outcomes with ORIF of condylar fractures in adults and children since the 1990s.

Kamath et al. performed a review on pediatric condylar trauma. They claimed that open surgery causes growth disturbance due to surgical manipulation and hinders mandibular development because of the damage and disturbance of the condylar growth centre [76]. They recommend avoiding ORIF in children under 12 years because of significant growth disturbance of the mandible [76]. The authors of the present review disagree with this view as it is not substantiated. Kamath et al. agreed with the great majority of the Anglo-American literature that condylar fractures in children should always be treated conservatively, as surgery creates a scar and, inevitably, growth restrictions. The authors of the present review believe that bad surgery can certainly cause heavy scars and a lot of damage, but this is not the case for an expert approach, as reported in the German literature, which they did not list among their references.

Bansal et al. compared ORIF and closed treatment outcomes in children with mandibular fractures [77]. They chose functional protocols over IMF and open surgery because of concern about growth disturbance caused by osteosynthesis and recommended nonsurgical management as the mainstay option for pediatric mandible fractures [77].

Kao and colleagues reported on the management of mandibular fractures of 150 children in a US tertiary-care hospital, where facial trauma was managed by ear, nose and throat specialists, plastic surgeons, and maxillofacial surgeons. They claimed that “compared with ORIF, MMF confers a decreased risk of long-term facial deformity” [58]. The authors of the present review consider such a claim baseless. Kao et al. resorted to rigid intermaxillary fixation with wires for two weeks and considered open surgery and osteosynthesis risk factors for growth restriction on the mandible [58]. Kao et al. described a setting where ear, nose and throat surgeons, plastic surgeons, and maxillofacial surgeons rotate in treating facial trauma, which is not ideal as the caseload becomes thin across three specialties. They wrote that “compared with ORIF, MMF confers a decreased risk of long-term facial deformity”, which is illogical, as the fractures that are treated conservatively with MMF are generally non- or minimally displaced, whereas the fractures treated with surgery are heavily displaced, and so they are entirely different categories that cannot be compared. They resorted to IMF with wires, which is a poor choice, and stated that surgery per se causes growth restriction, which is not the case.

In mainland Europe, maxillofacial surgeons treat facial fractures. The authors of the present review consider them the most appropriate specialists for condylar fractures due to their familiarity with this region and with the occlusion. Nevertheless, ultimately, the individual surgeon’s expertise matters more than his or her background.

Bruckmoser and Undt reviewed the literature concerning the management and outcome of condylar fractures in children and adolescents and concluded that nonoperative treatment usually yields satisfactory to excellent clinical results [78]. However, in adolescents, the outcome was often less favourable [78].

Theologie-Lygidakis et al. conducted a retrospective study on nonsurgical management of condylar fractures in children [79]. They recommended closed management as a default option unless in case of severe dislocation, projectile injuries and polytrauma, highlighting the importance of active kinesiotherapy to be carried out regardless of the type of management [79].

Recently, Esposito and colleagues performed a systematic review of the literature concerning the surgical treatment of pediatric mandibular condyle fractures [7]. They pointed out that the follow-up studies of dislocated condylar fractures demonstrated that facial asymmetry reduced the range of movement with shortened hypoplastic condyles and asymmetrical mandibles [7]. Open surgery ideally returns the condyle to its pre-injury position, allowing quicker functional recovery and better remodelling. Furthermore, open treatment of dislocated condylar fractures yielded satisfactory results in 87% of patients, clinically and radiographically [7]. On the other hand, severely displaced and dislocated condylar fractures treated conservatively showed a higher incidence of growth anomalies, asymmetry, malocclusion, masticatory dysfunction with pain clicking, and ankylosis [7]. Therefore, it was concluded that surgical treatment can lead to good or excellent results for severe dislocated and displaced condylar fractures in children and is superior to closed treatment [7]. Esposito et al., not based in an English-speaking country, demonstrated a logical approach, stating that repositioning the displaced condyle to its original position helps it immensely to remodel and heal. They reported a high prevalence of satisfactory results with open surgery based on imaging and function and highlighted that asymmetry, pain and ankyloses affect dislocated condylar fractures treated conservatively, which, of course, makes sense. Therefore, the authors of the present review agree with their perspective.

Over the past twenty years, there has been a growing preference for open surgery in children in mainland Europe, aligning with a previous similar trend for adult patients, based on suboptimal results after nonoperative management and better functional results with ORIF. Cornelius et al., in 1991, analysed retrospectively 65 patients between two and twelve years treated conservatively and with a follow-up between two and fifteen years. They found sixty-two patients with incomplete remodelling, varying degrees of discomfort and dysfunction, and only three with restitutio ad integrum [80].

Rasse in Austria and Neff in Germany pioneered this field in the 1990s, reporting consistently superior functional results in their patients treated with ORIF, even in the most challenging intraarticular fractures [51,81,82].

In those years, the internet was not yet as widespread as it is today and their articles, written in German, did not have the type of global diffusion that is possible nowadays for open-access articles written in English.

Hlawitschka et al. compared the results of open and closed treatment of condylar fractures in adults [83]. The ORIF group achieved better radiological results regarding the ramus height and changes to the condyle compared to the group managed with closed functional treatment [83]. Furthermore, after open treatment, the temporomandibular joint displayed significantly fewer irregularities in the condylar paths [83]. They concluded that open surgery improves function with a postoperative therapeutic exercise program [83].

The shift towards open surgery in the adults was corroborated by two seminal randomised controlled trials providing further evidence of the superiority of open reduction and internal fixation (ORIF) over closed treatments, not only for severe displacement or dislocation, but also for moderately displaced condylar fractures, which were defined as a deviation of 10 to 45 degrees or shortening of the ascending ramus by more than 2 mm [84,85]. Two meta-analyses in 2015 confirmed ORIF superiority versus closed treatment in adults [66,86].

To our knowledge, Rasse et al. wrote the first report describing ORIF for condylar fractures in children [87]. They retrospectively reassessed 37 children following surgical (No. = 25) or conservative (No. = 12) management of condylar fractures. Despite the worst starting point of the patients treated with open surgery, the morphological and functional outcomes were comparable. No adverse effects or complications resulting from the surgery, such as facial nerve paralysis, growth impairment, or prominent scars, were observed.

In children, Landes et al. favoured ORIF for displaced and dislocated condylar fractures to achieve “anatomical reduction, occlusal stability, rapid function, maintenance of vertical support, avoidance of facial asymmetry, less postoperative temporomandibular joint disorder incidence and no maxillomandibular fixation” [44]. They concluded, “Displaced and dislocated fractures treated with ORIF have fewer incidences of facial asymmetry, locking, and occlusal imbalance” [44].

According to Schiel et al., in cases with severe displacement (>45 degrees of deviation) and the dislocation of condylar base or neck fractures, a normal temporomandibular joint function cannot be achieved with nonoperative management, even in the pediatric patient [88].

More recently, Vesnaver questioned the universal validity of the conservative dogma, describing a retrospective series of pediatric patients treated with ORIF with excellent results concerning restoring normal anatomy—including the capsule and the disc—a complete range of motion and symmetric mandibular growth [61].

In China, Zhang et al. demonstrated favourable clinical and radiographic outcomes in pediatric patients treated with ORIF without surgical complications and achieved good TMJ function and facial symmetry [89].

After a dislocated condylar fracture, adaptations must happen at three levels—neuromuscular, skeletal, and dental—for the masticatory system to return to its pre-injury status [40].

An anatomic repositioning of the hard and soft tissues will reduce the need for substantial remodeling and create the best possible prerequisite for a restitutio ad integrum [29,40,42,51,89]. However, the least possible amount of surgical trauma should be inflicted on the tissues to minimise the disruption of the vascular support and scar formation. Therefore, this surgery is not for heavy-handed surgeons or novices [61].

Hofmann and colleagues conducted a retrospective analysis of 91 children and adolescents with mandibular fractures [90]. They operated on displaced and dislocated condylar neck fractures through an extra- or a transoral approach depending on their height, removing the plates routinely to prevent complications or growth disturbances after complete bone healing. Condylar head fractures were managed with functional protocols, including using orthodontic appliances [90].

Charcanovic reviewed the treatment of diacapitular fractures in children and adults, comparing open versus closed treatment [91]. He suggested ORIF for adult patients with fractures of the lateral pole and reduction of the mandibular height. On the other hand, nonoperative treatment was indicated for nondisplaced fracture, the major fragmentation of the condylar head making osteosynthesis impossible, and for all the intraarticular fractures in children regardless of their configuration [91].

He emphasised the importance of disc repositioning in open surgery to prevent ankylosis and preservation of the attachment of the lateral pterygoid muscle to avoid disruption of the circulation in the proximal fragment and to allow the recovery of the full range of motion in conjunction with physiotherapy [91].

In mainland Europe, some units treat surgically displaced non-comminuted condylar head fractures involving the lateral pole with ramus height-loss also in growing patients [10,44,61,85].

Many authors agree that surgery does not affect growth if it is done with a delicate, respectful approach to keep surgical trauma to a minimum [40,52,61,70,87,92].

Careful dissection around the condyle in ORIF does not compromise vascular support or cause condylar resorption [29].

For fractures with dislocation, ORIF has a low incidence of complications and is indicated even in children if the surgeon has adequate training [40,47,61,70].

Facial nerve injury is rare and primarily temporary in experienced hands after ORIF [29,61].

In the case of severe displacement (>45 degrees of deviation) and the dislocation of condylar base or neck fractures, ORIF is indicated even in small children, provided a surgeon with adequate know-how performs it [61,88].

Furthermore, in a high-energy injury, the disc, the capsule, and the ligaments are also torn and displaced; only with open surgery can they be repositioned and repaired [44,51,61,89]. With nonsurgical treatment, the above soft tissues will undergo scar retraction in malposition, leading to clicking, deviation and deflection, limited mouth opening, and further degenerative TMJ changes over the years [47]. Disc displacement is a precondition for ankylosis if bleeding bone surfaces come in contact [51].

If a sufficiently stable osteosynthesis is not achievable, no hardware should be left in situ to avoid iatrogenic complications, and a conservative treatment should be chosen instead as a fallback option [47].

At the first author’s institution, condylar head fractures are reduced and stabilised with two positional screws in dentate patients when the lateral pole is affected by a reduction of the mandibular height, and there is no major fragmentation, allowing for osteosynthesis [93]. Beneficial technical advancements to enhance the outcome quality are, among others, 3D printing and intraoperative CT scans, which have been described in detail elsewhere [93]. The size of the bone model, which is identical to the patient’s bone, gives the surgeon an appreciation of the feasibility of osteosynthesis in children and helps in the decision-making for or against open surgery [93].

The endoscopic-assisted transoral approach allows ORIF for condylar base and neck fractures, with the additional advantages of further reducing the risk of facial weakness and without external scarring, as demonstrated in adult and pediatric patients in his pioneering papers by Schön et al. and later confirmed by Schiel et al. in the pediatric population [47,88,94,95,96,97,98,99].

Recently, Abdelazeem described a series of endoscopically assisted transoral approaches for ORIF and hardware removal in pediatric patients, highlighting the advantages of this technique [100].

The drawbacks of the endoscopic-assisted approach are the need for special tools and the different skills from the one required for open surgery, the longer duration of the procedure and the limited indication of condylar base fractures with lateral displacement.

### 4.7. Titanium vs. Resorbable Hardware and Hardware Removal

Titanium hardware is chosen over resorbable polylactic acid and polyglycolic acid screws and plates by most authors because of its superior mechanical properties, ease of handling, biocompatibility and the possibility of removing it entirely [10,18,47,63,89,101]. On the other hand, resorbable plates and screws have larger dimensions, which are unfavourable in small children, have the worst mechanical properties, and can be inserted only after predrilling and pre-tapping [101]. Furthermore, plate bending and screw insertion are time-consuming and more complicated compared with titanium [101].

In a recent multicentric study involving 14 maxillofacial departments worldwide, out of a total of 82 pediatric patients with condylar fractures treated with ORIF, 95% of the condylar fractures were fixated with titanium hardware and 5% with resorbable plates and screws [10].

Rigid fixation on the growing calvarium caused growth restriction in the animal model [26]. However, there is no evidence of plate migration and growth restriction in pediatric mandibular fractures [18,26,56,63,102].

In a series of 96 children treated with 375 titanium plates and 1944 screws at several craniofacial localisations, Berryhill et al. reported a relatively small number of complications despite not removing the hardware unless specifically indicated [26]. Their overall rate for reoperation was 8%, with five cases of delayed growth and one of restricted growth at the cranial vault of a syndromic patient [26].

In a recent retrospective study on mandibular condyle fractures in children and adolescents, 82 patients had ORIF treatment, and 15 patients had their hardware removed (18%) [10].

For children over 12 years old, hardware was not removed in 97% of the cases, following expert recommendation [47]. However, in deciduous and mixed dentitions, plate removal was primarily planned (11 out of 15 cases), possibly due to concerns about a hindrance to the growth and the displacement of plates caused by the growth of new bone [10,101]. Plate removal necessitates a second hospital admission and general anaesthesia, and in cases of extraoral access, there is a risk of damaging the branches of the facial nerve.

Incidentally, Cremona et al. focused on plate removal after ORIF in non-condylar fractures in 383 children and adolescents in the same multicentric study [103]. They found that it was carried out only in symptomatic cases and not routinely [103].

Vercruysse et al. conducted a review on indications and complications of titanium osteosynthesis in pediatric maxillofacial trauma and concluded that it is a reliable and suitable treatment with few complications [104].

However, specifically, in the case of screw osteosynthesis for condylar head fractures, Skroch, Neff et al. observed partial condylar head resorption in 30% of their cases due to a stress-shielding effect. Therefore, they routinely remove the screws in their patients and recommend routine screw removal in this area [105].

Stress shielding refers to the reduction of usual mechanical demands on the bone due to the presence of an implant, leading to osteopenia as a functional adaptation.

Skroch recommended it at around six months postoperatively [105].

Johner, Neff and colleagues measured 3D bone resorption after condylar head fractures in adults and found it to be approximately 15% during the first six months postoperatively, recommending screw removal after six months to prevent the risk of protruding implants [106].

Smolka, Cornelius et al. also measured 3D bone resorption after ORIF for mandibular condylar head fractures in adults and reported minimal condylar resorption. Therefore, screw and plate removal was performed only in cases of loosening or complications [107].

Smolka et al. treated 48 patients surgically with a total of 55 condylar fractures. They observed no wound infection, dehiscence, salivary fistula, or facial nerve weakness. Screw and plate loosening was noted in four fractures (7%) [107].

Wheeler and colleagues routinely removed titanium plates and screws in the pediatric patient to prevent long-term growth disturbance and to allow ease of removal before they become encased in bone [108].

Hardware is removed only in selected cases at the first author’s institution.

Titanium plates and screws can be removed after six months to prevent possible complications after ORIF in children and adults. However, this implies hospital admission and general anaesthesia and entails the risk of a surgical procedure.

Alternatively, a follow-up with imaging is indicated to intercept significant resorption of the condylar head and avoid complications due to protruding implants. Drawbacks are hospital visits and radiation exposure.

If a sufficiently stable osteosynthesis is not achievable, no hardware should be left in situ to avoid the above iatrogenic complications, and a conservative treatment should be chosen instead as a fallback option [47].

### 4.8. Soft Tissue Injuries and Repair

When discussing condylar fractures, one tends to forget the concomitant injuries to the surrounding soft tissue structures, such as the muscles, capsule, ligaments, and disc [61]. Generally, surgeons focus on the bone, as it is detailed in CT scans and 3D reconstructions. Magnetic resonance tomograms (MRT) are not usually part of the acute work-up in trauma patients.

Capsule lacerations always accompany dislocations [44,51]. The disc is fixed very well to the condyle and, in case of dislocation, generally follows the proximal fragment, and it is generally repositioned in the fossa after fracture reduction during ORIF [51].

In cases of intraarticular fractures, after fracture reposition and osteosynthesis, the position of the disc can be controlled directly, and the capsule should be sutured if possible [51].

Fracture reposition and fixation also return the lateral pterygoid muscle to its original length and trajectory, allowing the return to preinjury mandibular excursions [51].

Liu and colleagues observed that anteriorly displaced discs in children may return to their normal position following closed treatment. This effect is due to the excellent elasticity of the tissue in children [109]. However, this is not the case in adults, where rupture of the posterior attachment of the disc is more likely to occur, and hence, the disk will stay in its displaced position. Prolonged anterior disc displacement was associated with poor condylar remodelling [109].

Zheng et al. examined 160 patients with 222 injured joints with a CT scan and an MRI preoperatively. Unsurprisingly, intracapsular condylar fractures were more likely associated with displaced discs than neck or condylar base fractures [110].

Soft tissue injuries associated with intracapsular condylar fractures include hemartrosis, disc displacement, retrodiscal tissue and capsular tears [111,112].

Severe injury to the articular disk and capsule is a significant factor in developing complications after nonoperative treatment of condylar fractures [113].

Yang et al. focused on the MRI examination of TMJs treated with close reduction after condylar fracture. They showed “antero-medial displacement of the articular disc, elongation and thickening of the retro tissue, and reactive bone formation of the condylar head”. Furthermore, “the presence of a portion of disc between the residual condyle and the fossa prevented the development of osteoarthritis and ankylosis”. On the other hand, “perforation of the bilaminar tissue and contact between the residual condyle and the fossa promoted osteoarthritic changes and ankylosis” [114].

### 4.9. Perioperative Antibiotics in ORIF

A recent multicentric retrospective review on pediatric condylar trauma reported the use of antibiotics for a week or longer in 94% of pediatric patients treated expectantly with IMF or ORIF [10]. Antibiotics are not indicated for closed fractures, only in open fractures, and should be continued until 24 h postoperatively [115,116]. Antibiotic prophylaxis beyond 24 h is associated with antibiotic resistance and side effects without any additional patient benefit [115,116].

### 4.10. Considerations for Clinical Practice

Some helpful considerations for the clinical practice are summarised below in table format (Table 1).

## 5. Future Directions

Multicentric randomised controlled trials with higher patient numbers, 3D radiologic measurements, validated functional indexes, and long-term follow-up could help clarify fracture-related and patient-related elements to indicate the most effective treatment protocol. However, these trials are very labour-intensive. Nevertheless, recent advances in artificial intelligence could help reduce this mammoth task.

In the future, MRI imaging could become quicker and cheaper and find application in the work-up of the acutely injured patient, providing direct visualisation of type and degree of soft tissue damage, helping with the decision-making (conservative vs. operative treatment) and helping during open surgery.

## 6. Conclusions

Nondisplaced and minimally displaced fractures should be treated expectantly; severely displaced non-comminuted fractures can be safely operated on if the expertise is available, even in patients with deciduous dentition. MMF should be foregone in children due to its many drawbacks. Functional protocols can achieve excellent outcomes, especially in deciduous dentition.

## Figures and Tables

**Table 1:** Considerations for clinical practice.

1.	In many cases, nonsurgical and surgical treatment can lead to successful outcomes if the clinician has adequate know-how [42,47,70,92].
2.	An interdisciplinary consultation with an orthodontist could be beneficial in exploring the potential of conservative protocols [46].
3.	The informed consent must acknowledge the possibility of functional and surgical treatments.
4.	Expectant treatment is the best option for fractures without dislocation or minimal displacement. It is minimally invasive and highly successful. Condylar remodelling provides an extraordinary example of a natural feature.It is fair to say that different rules apply to pediatric maxillofacial trauma than the ones that hold for adult patients.
5.	For fractures with dislocation, ORIF has a low incidence of complications and is indicated even in young children if the surgeon has adequate training [40,47,61,70,92].
6.	Concentrating pediatric fractures in a few high-volume centres would be desirable, as this would allow the development of adequate expertise through a sufficient caseload.
7.	Fracture height: The lower and the more dislocated the mandibular condyle fracture, the more ORIF is indicated. The higher and the less dislocated the mandibular condyle fracture, the more expectant or functional treatment is indicated.
8.	Consider an endoscopic transoral approach for condylar base fractures with lateral displacement [47,97,99].
9.	Many experts treat children older than 12 as adults concerning ORIF indications [47].
10.	In the age group between six and twelve, opinions diverge between different authors on treatment modality [47].

## Data Availability

No new data were created or analyzed in this study. Data sharing is not applicable to this article.
